# Cerebral Vasculitis in a Case of Meningitis

**Published:** 2017

**Authors:** Devdeep MUKHERJEE, Agnisekhar SAHA

**Affiliations:** 1 Department of Pediatric Medicine, Institute of Child Health Kolkata, India.; 2 Department of Pediatric Medicine, Fortis Hospitals, Anandapur, Kolkata, India.

**Keywords:** Meningitis, Cerebral vasculitis, Methylprednisolone (MP)

## Abstract

Cerebral vasculitis is a serious complication of meningitis with *Streptococcus pneumoniae. *We report a 5 yr old girl who was admitted in May 2015 at Fortis Hospital, Kolkata, India and was diagnosed to have pneumococcal meningitis with vasculitis on DWMRI within a week of onset of fever. She was given high dose of methyl-prednisolone simultaneously with antibiotics and successfully treated without any neuro-sequelae. Although vasculitis has been documented to develop as sequelae to bacterial meningitis usually in the second week, our patient had an early presentation. Treatment with high dose steroid along with antibiotics resulted in dramatic improvement with no neurodeficit. DW-MRI is an important investigation to pick up early, evidence of vasculitis in a patient with meningitis who is showing neurological deteriorations despite improvement in blood parameters.

## Introduction

Cerebral vasculitis is a serious complication of meningitis with *Streptococcus pneumoniae*. We report a 5 yr old girl admitted in May 2015 at Fortis Hospital, Kolkata, India. She was diagnosed to have pneumococcal meningitis with vasculitis on DWMRI within a week of onset of fever.

## Case Report

A 5 yr old girl was admitted to the Pediatric Intensive Care Unit (PICU) with a history of fever for 5 d along with vomiting and obtunded sensorium for 24 h. On admission, she had a temperature of 103.4 F. She was disoriented, irritable and had photophobia. Her pupils were symmetrical in size and reacted well to light. Kernig”s sign was positive. Her chest was clear and she had a heart rate of 122/min. Her blood pressure was 104/64 mm of Hg. She did not have any features of shock. She had a Glasgow coma score of 9/15 (Eye opening to pain, inappropriate words, and flexor response).

She had not been vaccinated with the pneumococcal vaccine. However, all others had been administered as per age according to the Indian Academy of Pediatrics Vaccine schedule.

On admission, she had hemoglobin of 11.1 gm/dl. Total leucocyte count (TLC) was 20600 /cumm (Neutrophils 79%, lymphocyte 7%) and C reactive protein (CRP) was 311.51 mg/L (normal <5). Her electrolytes, liver function, renal function tests, arterial blood gas, capillary blood glucose and chest x-ray were normal. Suspecting meningitis, we started treatment with intra venous (IV) fluids, IV ceftriaxone and vancomycin. Dexamethasone was also administered prior to the first dose of antibiotics.

**Fig 1:**
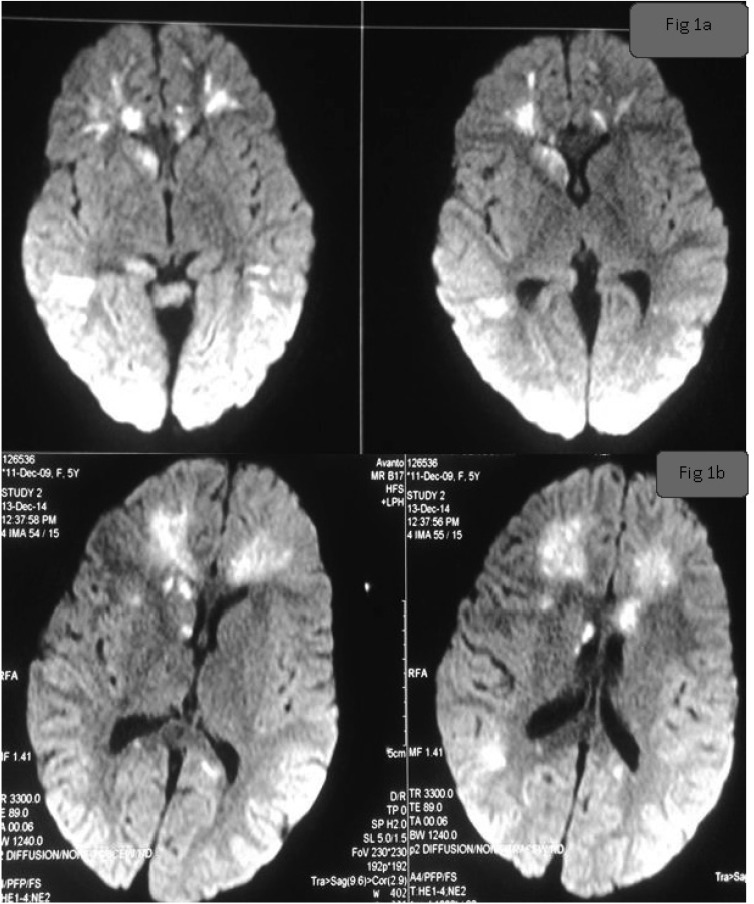
**a**) Diffusion weighted (DW) MRI was suggestive of meningitis with multiple bilateral small subcortical,

Overnight the child did not show any significant improvement and a decision to do a Magnetic Resonance Imaging (MRI) of the brain was planned on the following day. Diffusion weighted (DW) MRI was suggestive of meningitis with multiple bilateral small infarcts possibly secondary to small vessel vasculitis (Figure 1a). Cerebro spinal fluid (CSF) had a cell count of 120 with neutrophils 70% and lymphocytes 30%. Protein was 143 gm/dl and sugar was 38 gm/dl (blood sugar 118 gm/dl). 

CSF gram stain showed Gram-positive diplococci. Subsequently, both blood and CSF culture grew pan-drug sensitive *S. pnemoniae.* By the fourth day, TLC had decreased to 11800/ cmm and CRP to 36.1 mg/L. There was a decrease in the fever peak, with an increase in the interval. However, her sensorium continued to deteriorate and on the 4^th^ day, it was significantly worse. Repeat DWMRI showed areas of diffusion restriction in bilateral temporal, frontal and parietal regions. Lesions of both caudate nucleus and corpus callosum had become larger and more confluent. There were few new lesions noted in the corpus callosum anteriorly and in the left frontal white matter (Figure 1b). Although there was an improvement in inflammatory parameters as evidenced by decrease in TLC and CRP, there was a clinical deterioration as evidenced by rapid progression in vasculitis on MRI. Pulsed methylprednisolone (MP) was administered from day 4. Anti nuclear antibody, pANCA, cANCA and complements (C3 and C4) were negative.

IV MP was given at 30 mg/kg/day for 5 d following which steroid was given as oral dexamethasone. Two days after starting MP, she began to show gradual improvement of sensorium. Feeding was gradually established and she was shifted out of PICU. She was discharged after getting IV antibiotics for 14 days. Oral dexamethasone was tapered over 4 wk. At 3 months follow up, she was well with no neurological sequelae. Informed consent from parents and ethical clearance from Institute ethics committee was duly obtained.

## Discussion

Invasive pneumococcal sepsis causes high morbidity and mortality in the pediatric population. Pneumococcal meningitis is known to cause various sequelae such as vasculitis, subdural empyema, brain abscess and non-communicating hydrocephalus (1). In a study on 221 children, 42% developing complications and sequelae were found (2). However, the incidence of pneumococcal sepsis is much less in vaccinated children. Our index case, not immunized with pneumococcal vaccine, was diagnosed to have *S. pneumoniae* from both blood and CSF culture.

Pneumococci rapidly undergo multiplication and release immunogenic products, causing an inflammatory response. Bactericidal antibiotics may further accentuate this process (3). Bacteria penetrate the vascular wall causing vasculitis characterized by arteritis, infarction, thrombosis, thrombophlebitis and vascular narrowing. This usually presents in the second week, with an initial clinical improvement followed by neurological deterioration (4-5). Our child, however, continued to deteriorate and did not show this typical two-phase progress. The fever and inflammatory markers did improve; the antibiotics were controlling the infection. This was also expected, as the organism was sensitive to the antibiotics given. Therefore, the worsening (documented clinically and radiologically) was possibly due to an immunological event rather than infection per se.

DW-MRI is a sensitive imaging technique to detect early changes in vasculitis compared to T2W or Fluid Attenuation Inversion Recovery (FLAIR) sequence. On DW sequence, the lesions were both more numerous and conspicuous in our patient. Hemorrhagic and ischemic areas, periventricular white matter lesions, parenchymal and meningeal enhancements can be seen as complications of meningitis. Vasculitis, characterized by narrowing of vascular lumen occurs in the acute phase of meningitis. These changes vary with the phase and severity of infection (6-8).

Cerebral vasculitis should be treated with high dose pulse MP therapy (3 to 5 d) followed by tapering doses of oral prednisolone. Cyclophosphamide can also be added to the treatment protocol (3, 9-12). As our child was deteriorating and MRI was getting worse because of the gradually progressive vasculitis, we decided to initiate MP early along with ongoing antibiotics, to which the child responded dramatically. We followed up the MP with a tapering dose of oral prednisolone for 6 wk to our patient responded well. Although there is no available literature on the simultaneous use of steroids with antibiotics, we were successful in curing our child.

There should be a high index of suspicion for vasculitis in pneumococcal meningitis. It can cause temporary or permanent neurological sequelae (10). Performing DWMRI early, if there is suspicion, with prompt initiation of high dose steroids can be associated with favorable outcome, as we saw in our patient. 

callosal and right caudate nucleus infarcts possibly secondary to small vessel vasculitis

b) Repeat DWMRI showed areas of diffusion restriction in bilateral temporal, frontal and parietal regions. Lesions of both caudate nucleus and corpus callosum had become larger and more confluent. There were few new lesions noted in the corpus callosum anteriorly and in the left frontal white matter

**In Conclusion,** vasculitis has been documented as sequelae to bacterial meningitis. However, it usually presents in the second week, unlike the early presentation is seen in our patient. DW-MRI is an essential tool to pick up early evidence of vasculitis in a patient with meningitis who is showing neurological deterioration, despite improvement in blood parameters. Vasculitis if treated early can minimize the chances of neuro sequelae significantly, as we saw in our patient. 

## Notes

**Authors’ contribution: **Both the authors were involved in patient management, data collection and interpretation of data. Both were involved in drafting of manuscript. Draft has been approved by both authors prior to submission and both authors are accountable for all aspects of the work.
